# Thermal Characterization of Conductive Filaments in Unipolar Resistive Memories

**DOI:** 10.3390/mi14030630

**Published:** 2023-03-10

**Authors:** Cristina Aguilera-Pedregosa, David Maldonado, Mireia B. González, Enrique Moreno, Francisco Jiménez-Molinos, Francesca Campabadal, Juan B. Roldán

**Affiliations:** 1 Departamento de Electrónica y Tecnología de Computadores, Facultad de Ciencias, Universidad de Granada, Avd. Fuentenueva s/n, 18071 Granada, Spain; 2 Institut de Microelectrònica de Barcelona, IMB-CNM (CSIC), Carrer dels Til·lers s/n, Campus UAB, 08193 Bellaterra, Spain; 3 Departamento de Física y Matemáticas, Facultad de Ciencias, Universidad de Alcalá, Pl. de San Diego s/n, Alcalá de Henares, 28801 Madrid, Spain

**Keywords:** resistive switching memory, RRAM, temperature characterization, simulation, variability, modeling, kinetic Monte Carlo, series resistance

## Abstract

A methodology to estimate the device temperature in resistive random access memories (RRAMs) is presented. Unipolar devices, which are known to be highly influenced by thermal effects in their resistive switching operation, are employed to develop the technique. A 3D RRAM simulator is used to fit experimental data and obtain the maximum and average temperatures of the conductive filaments (CFs) that are responsible for the switching behavior. It is found that the experimental CFs temperature corresponds to the maximum simulated temperatures obtained at the narrowest sections of the CFs. These temperature values can be used to improve compact models for circuit simulation purposes.

## 1. Introduction

Resistive memories, also known as resistive random access memories (RRAMs), are attracting interest from the scientific community and the industry in the last twelve years as a consequence of their great potential for a wide variety of applications of interest in the electronics landscape [1]. RRAMs can be used as non-volatile memories [2]; several companies commercially use these devices (TSMC for its 40 nm [3], 28 nm [4], and 22 nm [5] nodes; INTEL for its 22 nm [6] node). Another great field of development is linked to neuromorphic engineering [2,7,8,9,10,11,12,13,14,15,16]. In addition, these emerging devices are interesting for hardware cryptography in the context of the Internet of Things ecosystem [17,18,19,20]. Moreover, these devices, and other memristive devices [21], can be employed as radio-frequency switches for mobile communications [1].

RRAM operation is based on resistive switching (RS), whose origin is related to ion movement and concurrent redox reactions inside the dielectric and at the dielectric/electrode interfaces [22,23]. These devices present an outstanding endurance (above >10^10^ cycles [24]); they have demonstrated a good speed (<10 ns) and a large resistance window [1]. Their fabrication is compatible with CMOS technology and the devices can be built in compact crossbar structures [2]. RRAMs can be operated with very low power consumption. Resistive memories can be employed in a digital context, where they show two stable resistive states: the high resistance state (HRS) and the low resistance state (LRS); moreover, an analog perspective is appropriate for neuromorphic computing applications [1,7], although their usually exhibited non-linear response could make the weight update more difficult [25]. However, even devices that exhibit abrupt (digital) switching between the HRS and LRS can still be used for the implementation of binarized neural networks [26] or binary weight spike-time-dependent plasticity (STDP) learning rules [27].

The device operation depends on the dielectric and electrode materials. Different types of RRAMs are distinguished depending on the RS physical mechanisms. For filamentary conduction, which is related to the creation and destruction of conduction paths (conventionally designated as conductive filaments, CFs), the two main device types are those whose CFs are formed by dielectric regions with a high concentration of oxygen vacancies (known as valence change memories [23,28]), and those where the CFs are formed by paths rich in metallic atoms, which migrate from an active electrode. We consider here this latter type of devices, including atomic layer deposition-grown HfO_2_ dielectrics [29] that show filamentary conduction of metallic-like CFs [30,31]. The random nature of the CF formation leads to a strong variability [32,33], although techniques for controlling the CF size and location are subjects of research [32]. The CF temperature is critical to trigger the physical mechanisms behind RS [31]. Determining this temperature is a complicated issue both from the experimental [34] and theoretical [35] perspective. Although some measurements have been performed on hot spots in the electrodes, the key temperature for correctly describing the RS characteristics is at the CF narrowing [35], where Joule effects are higher and where, in general, the CF rupture takes place. In this respect, indirect strategies combining measurements with simulations are performed (mostly in the reset process, the transition from the LRS to the HRS, when the CF is broken) [34,36]. In order to deepen understanding of the issue, we present here a study to characterize the CF temperature and understand the role of this essential magnitude.

This manuscript is addressed as follows: in Section 2, we introduce the experimental details; in Section 3, the simulations are described; and in Section 4 the main results obtained are presented. Finally, the conclusions are drawn in Section 5.

## 2. Device Fabrication and Measurement Setup

**Figure 1:**
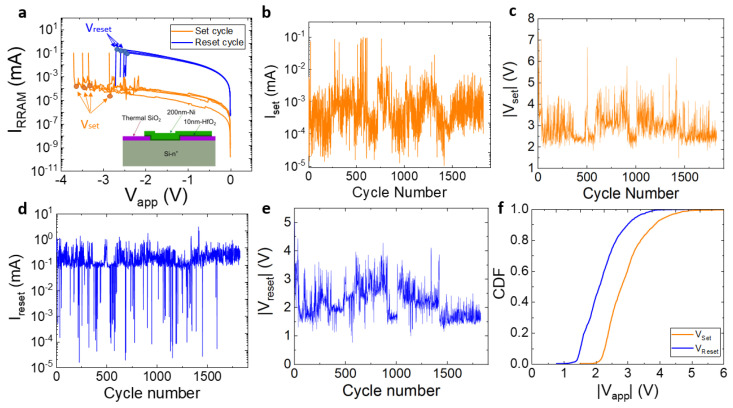
(**a**) Some experimental I-V curves of the devices under study for 1800 successive RS cycles in a long series depicting the set and reset voltages. The device structure is shown in the inset. (**b**) Set current, (**c**) set voltage, (**d**) reset current, (**e**) reset voltage versus cycle number, (**f**) cumulative distribution functions for the set and reset voltages.

The devices, based on the Ni/HfO_2_/Si stack, were fabricated on (100) n-type CZ silicon wafers (resistivity 0.007–0.013 Ω cm); see the schematics in Figure 1a [29]. The HfO_2_ dielectric layer (10 nm-thick) was grown by atomic layer deposition at 225 °C, using tetrakis (Dimethylamido)-hafnium (TDMAH) and H_2_O as a precursor, and the carrier and purge gas was N_2_. The top metal electrode (Ni with a 200 nm thickness) was deposited by magnetron sputtering and patterned by lift off. The device area was 15 µm × 15 µm.

The current–voltage (I-V) measurements were performed with a HP-4155B semiconductor parameter analyzer (Hewlett Packard, Palo Alto, California, United States). A negative voltage was applied to the top Ni electrode and the Si substrate was grounded. Successive I-V measurements for long RS series were obtained; the I-V meter was connected to the computer via GPIB (General Purpose Instrumentation Bus) and controlled using MATLAB (Matlab2020, Mathworks Inc, Natick, Massachusetts, United States). This allows us to automatically detect the reset current drop and subsequently stop the voltage ramp. Notice that a current compliance of 100 µA was employed during the set process. A total of 1800 successive RS cycles were measured; some I-V curves are shown in Figure 1a. The set and reset voltages and currents are plotted versus cycle number in Figure 1b–e. The cumulative distribution functions of the set and reset voltages are plotted in Figure 1f. The set and reset voltage were calculated by detecting current differences as described in MS2 and MR2 techniques in [37].

## 3. Device Simulation and Conductive Filament Temperature Determination

The simulation developed here is based on a finite difference 3D simulation domain of the memory cells described in Section 2, where the current and temperature are calculated accurately by solving the corresponding differential equations in a homogeneous approach of the materials included. One or several conductive filaments can be included in the simulation with cylindrical or truncated-cone shapes, whose geometries are calculated at every simulation step by considering the RS kinetics.

### 3.1. Simulator Description

The simulator describes the device electrical characteristics and the RS dynamics. The electrodes (we include 10 nm thick regions next to the dielectric) have been incorporated in the simulation domain (SD), which includes the dielectric. The grid consists of a set of 300 × 300 × 240 nodes. A uniform mesh with grid mesh distance of 0.125 nm was employed (the Ni atom radius approximately).

The 3D heat equation (Equation (1)) is solved to describe the device thermal distribution. Its solution is achieved by means of a fully explicit finite difference method [38]:

(1) $$\dot{q}=-\nabla \cdot \left[k_{th}\left(x,y,z\right)\nabla T\left(x,y,z\right)\right]$$

where *T*(*x*,*y*,*z*) is the temperature at each SD point, *k_th_*(*x*,*y*,*z*) is the thermal conductivity, and $\dot{q}$ stands for the energy generated per unit time and volume. Dirichlet boundary conditions were used at the outer electrode layer surfaces (room temperature was fixed at these outer points, a reasonable assumption taking the high electrode thermal conductivity into account); perfectly matched layers (PML) were used at the SD lateral faces [39]. The CF is assumed to be a truncated cone [40] with metallic-like conduction properties (the electric conductivity was 3 × 10^5^ (Ω m)^−1^ in line with values reported in previous works [41]). Joule heating takes place at the CF since the current is assumed to flow through the CF until it is ruptured [42]. The device series resistances are also included in the simulation tool. The heat generation rate ($\dot{q}$) is calculated by means of the electrical conductivity and the electric field distribution in the CF, where a linear increase of the resistivity is assumed as a consequence of the temperature rise (the temperature coefficient is taken as *α_T_* = 0.0005 K^−1^, as explained below). Three-dimensional thermal conductivities are employed in the electrodes (*k_th_*(silicon) = 148 W/(m K), *k_th_*(Ni) = 90 W/(m K)). The CF thermal conductivity was (*k_th_*(CF) = 11 W/(m K)), a reasonable value according to ref. [31]. For the oxide thermal conductivity, we use *k_th_*(HfO_2_) = 1.0 W/(m K), which is in line with the values reported in [43,44]. The numerical solution of Equation (1) was developed by means of the weighted residual method [45] in a particular derivation known as the control-volume formulation.

The CF radii evolve following Equation (2), where an average Arrhenius-like mechanism is assumed to control the variation of the high-concentration regions of metallic atoms that shape the CF and produce the set and reset processes [46]:

(2) $$\frac{dr\left(z\right)}{dt}=\pm A\cdot e^{-\frac{E_{a}}{kT\left(z\right)}}$$

where *r*(*z*) stands for the CF radius at the *z* coordinate, *A* is a fitting preexponential constant, *k* is the Boltzmann constant, and *E_a_* is the activation energy of the main physical mechanism behind the CF variation (we assume that the mechanism modeled in Equation (2) has the strongest influence in the CF kinetics; it could also be seen as a model that averages different mechanisms involved).

A summary of the simulation parameters is provided in Table 1.

**Figure 2:**
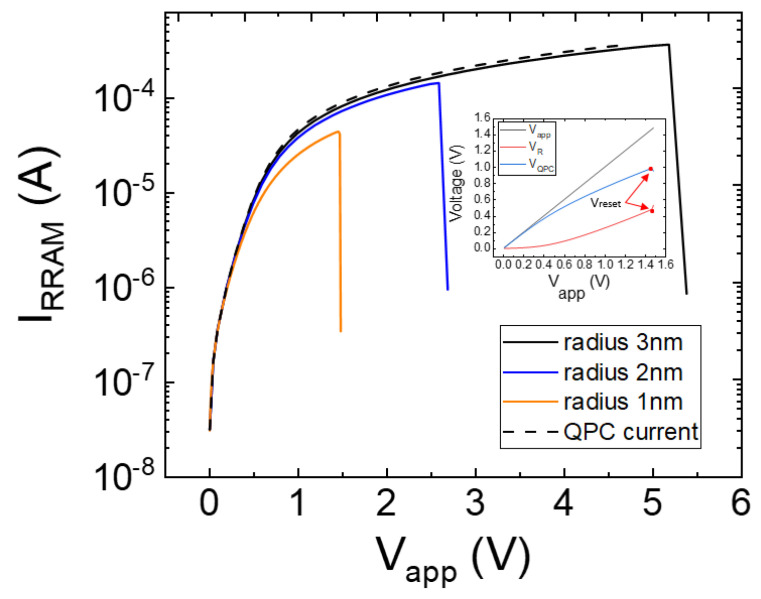
Simulated current versus voltage and description of the different components; see the voltage components in the inset (*V_QPC_* + *V_R_* = *V_app_*). Three CF radii are considered (1 nm, 2 nm, 3 nm) for cycles #1, #2, and #3 (inset: voltage curves for cycle #1). The QPC parameters are: α = 15 eV^−1^, *β* = 0.55, *Φ* = 0.26 eV, *N* = 2.

In each CF, a module is included to account for quantum effects due to a lateral filament constriction. These effects are modeled according to the quantum point contact (QPC) theory [47,48]. The appropriateness of this model, and the limitations and approximations needed to reach its final analytical form are discussed in depth in [49]. The QPC model current can be calculated as follows (Equation (3)) [47,48]:

(3) $$I=\frac{2eN}{h}\left\{eV_{QPC}+\frac{1}{\alpha }ln\left[\frac{1+e^{\alpha \left(\Phi -\beta eV_{QPC}\right)}}{1+e^{\alpha \left(\Phi +\left(1-\beta \right)eV_{QPC}\right)}}\right]\right\}$$

where *Φ* is assumed to be the potential barrier height measured with respect to the Fermi level, *α* is a parameter linked to the potential barrier thickness at the Fermi level, *V_QPC_* is the voltage that drops at the sides of the CF constriction (in a fraction of *β* and (1 − *β*), as suggested in [47]), *e* is the elementary electron charge, *I* is the CF current, and *N* is the number of active channels in the CF [47,48]. Accordingly, the constriction voltage drop, *V_QPC_*, is equal to the externally applied voltage to the device, *V_app_*, minus the voltage drop in the rest of the CF, *V_R_* (*R* stands for the CF resistance; it is an ohmic component). Notice that due to this and to the role of the series resistance linked to the electrodes, the resistance of the devices would be much higher than the contribution of the QPC model, which could be in some cases (very low barrier heights and *N* = 1) equal to 1/G_0_. Therefore, for our simulator the following equation stands for the I-V curve:

(4) $$V_{QPC}=V_{app}-V_{R}=V_{app}-I_{RRAM}\;R_{CF}$$

where *I_RRAM_* is the device current and *R_CF_* is the filament resistance. We simulated several I-V reset curves to assess the influence of the different components contributing to the current. For the sake of simplicity, we consider voltage absolute values henceforth. In Figure 2, the total current is plotted in solid lines, while the QPC current component (i.e., the QPC current assuming a null *R_CF_*) is shown in dashed lines (the QPC parameters are similar for all the curves shown). At low voltages, the I-V curve is mainly influenced by the QPC component; however, as the CF resistance rises, the ohmic component role increases, and the total current drops in comparison to the isolated QPC current component. Note that as the CF radius rises (a cylindrical CF shape is assumed here), the ohmic component influence decreases. As the current increases, the *V_QPC_* weight with respect to V_R_ in the total device voltage (*V_app_*) changes (see the inset in Figure 2). We simulated three different reset I-V curves for similar devices with cylindrical CFs with the following radii: 1 nm, 2 nm, and 3 nm. The I-V curves are identified as cycles #1, #2, and #3, respectively.

**Figure 3:**
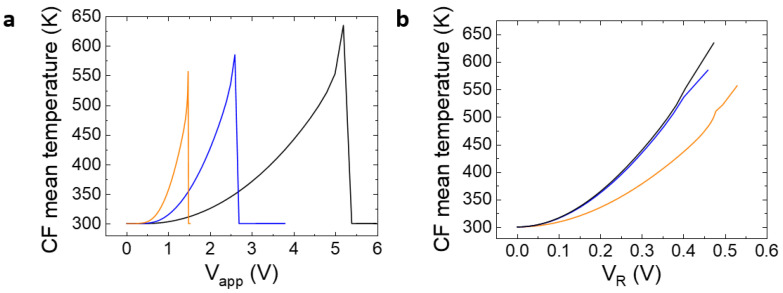
(**a**) Average CF temperature along the simulated I-V curves during the reset process versus the device applied voltage, (**b**) average CF temperature versus V_R_. Three CF radii are considered (1 nm, 2 nm, 3 nm) for cycles #1 (orange line), #2 (blue line), and #3 (black line).

The higher the CF radius, the lower the CF resistance. Notice that our model works well prior to the reset event; at this point, the CF is broken and there is an abrupt current decrease until a value that corresponds to charge conduction across the dielectric between the remaining filament tip and the electrode. The CF average temperature obtained for the simulated curves is shown in Figure 3.

See in Figure 2 that the reset voltage increases with the CF radius. A slightly higher temperature is obtained for higher radii due to the current increase (higher Joule effects), although the difference is not significant. That is why the approximation followed in simpler modeling approaches, such as those based on circuit breaker networks, employs a threshold value for the temperature that triggers the formation/rupture of the conductive filaments [50,51].

### 3.2. Experimental Conductive Filament Temperature Extraction

**Figure 4:**
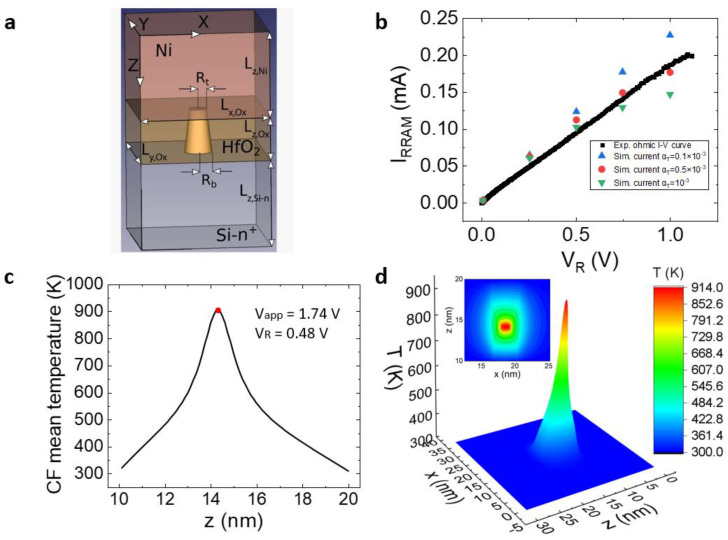
(**a**) Device schematic for the 3D simulations, (**b**) experimental ohmic I-V curve and simulated current for different temperature coefficients, (**c**) temperature distribution along the CF axis for *V_app_* = 1.74 V (0 < *z* < 10 nm corresponds to the Ni electrode (the CF/top electrode interface is placed at *z* = 10 nm)); the dielectric corresponds to 10 nm < *z* < 20 nm and the CF/bottom electrode interface is found at *z* = 20 nm. The maximum temperature is found in the [14 nm, 15 nm] interval due to the CF shape assumed, with the lower radius at the Ni/dielectric interface. (**d**) Three-dimensional temperature plot at the maximum temperature plane in the Y axis (inset: contour plot of the temperature distribution for the same X-Z axes cross-section).

The structure we simulate is shown in Figure 4a, where *R_t_* and *R_b_* stand for the CF top (Ni/HfO_2_ interface) and bottom (Si-n+/HfO_2_ interface) radii. Here, we obtain the temperature at each point of the simulation domain (Equation (1)); however, these data cannot be determined experimentally [34]. Therefore, a single temperature value is associated to the CF for each applied voltage for modeling purposes, which can be employed in simulators based on circuit breakers and in compact models. To shed light on this issue, a methodology to estimate the average conductive filament temperature is proposed, following [36]. A different perspective, although related to the one reported in [36], could also be employed for the average CF temperature extraction [52]. We attribute the CF resistance change in the low-voltage part of the reset I-V curves to the CF temperature increase (the CF shape is assumed to be fixed at these temperatures); see Equation (5) for the resistivity which determines the CF resistance for a fixed CF volume (in the LRS, we neglect charge conduction outside the CF):

(5) $$\rho_{CF}\left(T\right)=\rho_{CF0}\left[1+\alpha_{T}\left(T-T_{0}\right)\right]$$

Since we have a current point for each applied voltage, at most, we can estimate a CF temperature derived from the device resistance reduction. A correction to Equation (5) was proposed, adding a coefficient to account for the CF temperature nonuniformity, $\rho_{CF}\left(T\right)=\rho_{CF0}\left[1+\gamma \alpha_{T}\left(T-T_{0}\right)\right]$, with *γ* = 2/3 for a cylindrical CF [53]. The associated resistance can be calculated for truncated-shaped CFs and can consequently extract the CF temperature.

We first need the temperature coefficient that can be estimated by comparing experimental with simulation data, assuming a device such as the one described in Figure 4a. The QPC component was extracted from the simulation data and the experimental measurements and the ohmic component was analyzed (and compared) in both cases. Since the CF shape is supposed to be invariant until the last portion of the I-V curve, close to the reset voltage [53], the resistance variation is associated to thermal effects [35] (Figure 4b). In this manner, we are able to determine the temperature coefficient (*α_T_* = 0.0005 K^−1^) that works well in our simulation tool for the experimental data and the models we are considering here (we fitted the lower part of the I-V curve that presents a low temperature due to the low currents associated).

See a detailed CF thermal distribution along the dielectric length (Figure 4c). An average temperature was calculated for each CF slice along the CF length to plot this figure. A temperature cross-section in the X-Z planes is shown in Figure 4d. Nevertheless, with the experimental I-V curve, we just can obtain one CF temperature datum for each voltage point in the I-V curve.

## 4. Results and Discussion

**Figure 5:**
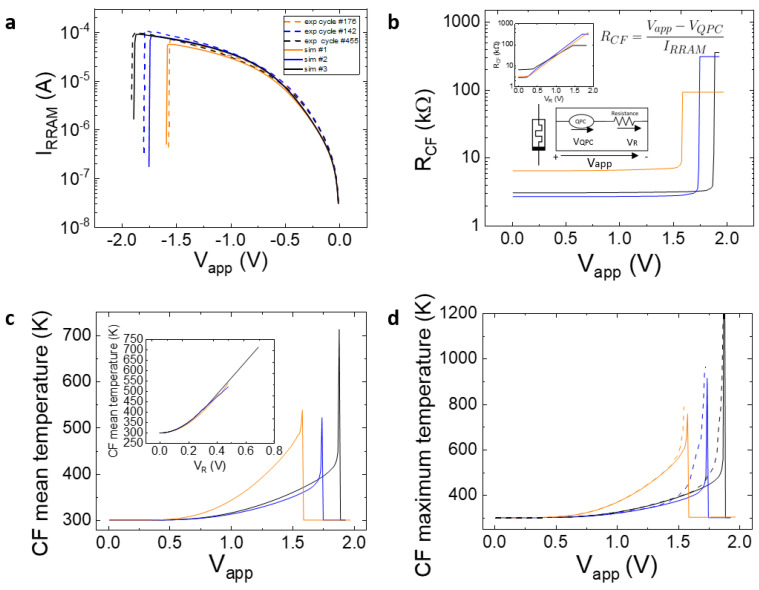
(**a**) Experimental (dashed lines) and simulated (solid lines) I-V curve of the devices under study. Truncated cone CFs of different radii are employed (radii: Sim #1 *R_t_* = 0.875 nm, *R_b_* = 1.75 nm; Sim #2 *R_t_* = 1.75 nm, *R_b_* = 2.25 nm y; Sim #3 *R_t_* = 1.50 nm, *R_b_* = 2.25 nm). (**b**) Ohmic resistance extracted for the simulated reset curve (inset: schema of the model employed and ohmic resistance vs. *V_R_*), (**c**) average CF temperature at each I-V point along the simulated curve (inset: average CF temperature versus *V_R_*), (**d**) simulated CF maximum temperature and the CF temperature (dashed lines) obtained experimentally with a process in line with Ref. [53].

We obtained the CF temperature by simulating I-V curves and fitting several experimental curves (see Figure 5a). In each case, the QPC model parameters and the CF shape were tuned. The ohmic resistance (*R_CF_*) component was extracted and plotted separately (Figure 5b); the simulated CF mean temperature was also calculated (Figure 5c). An average was calculated with the point-to-point distribution in the filament. Assuming the CF shape fitted in Figure 5a, *R_CF_* was calculated, and the experimental CF temperature (using Equation (5)) could be estimated (see the dashed lines in Figure 5d). See that the extracted temperature is closer to the CF maximum temperature (plotted in Figure 5d) than to the CF mean temperature (Figure 5c).

This result makes sense since the temperature at the CF narrowing (where the higher temperatures are produced by the current lines funneling) determines the CF rupture and formation that triggers the reset and set processes. We also calculated the temperature following Ref. [52] and found no significant differences with the results plotted in Figure 5d. As shown in Figure 5, the key CF temperature to consider from the compact modeling viewpoint is the maximum one found in the detailed simulation distribution. Therefore, the thermal resistances and capacitances to model the device temperature [35], which is assumed to be the CF temperature, have to be carefully chosen.

## 5. Conclusions

A new procedure to determine the temperature in RRAM conductive filaments is presented. We make use of experimental data of unipolar devices based on the Ni/HfO_2_/Si-n+ stack to estimate the operation temperature and the temperature coefficient of the device resistivity. A 3D RRAM simulator is developed that allows for the obtaining of the temperature distribution in the device’s active parts, in particular, in the conductive filaments that are created and ruptured to allow resistive switching. The CF temperature extracted is close to the maximum temperatures obtained by simulation at the CF narrowest sections. This temperature information is essential for the compact modeling of these emerging devices.

## Figures and Tables

**Table 1:** Physical parameters used in the simulations.

α*_T_*	0.5 × 10^−3^ K^−1^	CF thermal parameter
*k_th_*(Si)	148 W K^−1^ m^−1^	Si thermal conductivity
*k_th_*(CF)	11 W K^−1^ m^−1^	CF thermal conductivity
*k_th_*(Ni)	90 W K^−1^ m^−1^	Ni thermal conductivity
*k_th_*(HfO_2_)	1 W K^−1^ m^−1^	HfO_2_ thermal conductivity
σσ*_CF_*_0_	3 × 10^5^ Ω^−1^ m^−1^	CF electrical conductivity
*T*_0_	300 K	Room temperature
*E_a_*	0.84 eV	Activation energy

## Data Availability

The data presented in this study are available on request from the corresponding author.
